# Massive left ventricular Pseudoaneurysm four weeks post-myocardial infarction and percutaneous coronary intervention: a case report

**DOI:** 10.1093/omcr/omaf088

**Published:** 2025-07-14

**Authors:** Muhammad Umar Farooq, Shabana Kausar, Muhammad Fawad Tahir, Munaum Ali Khan, Adnan Nasir, Tanesh Ayyalu, Mohammad Hammad Jaber Amin

**Affiliations:** Department of Cardiology, Rawalpindi Institute of Cardiology, Rawal Road, Chaklala Cantt, 46000, Rawalpindi, Pakistan; Department of Cardiology, Rawalpindi Institute of Cardiology, Rawal Road, Chaklala Cantt, 46000, Rawalpindi, Pakistan; Department of Cardiology, Rawalpindi Institute of Cardiology, Rawal Road, Chaklala Cantt, 46000, Rawalpindi, Pakistan; Department of Medicine, HBS Medical and Dental College, Lehtrar Road, Tramari Chowk, 46000, Islamabad, Pakistan; Department of Cardiology, Rawalpindi Institute of Cardiology, Rawal Road, Chaklala Cantt, 46000, Rawalpindi, Pakistan; Department of Cardiology, Rawalpindi Institute of Cardiology, Rawal Road, Chaklala Cantt, 46000, Rawalpindi, Pakistan; Department of Cardiology, MedStar Georgetown University Hospital, 3800 Reservoir Rd NW, Washington, DC 20007, United States; Department of Medicine, Al Zaiem Alzahari University, Ahmed Qasim Street, 11111, Khartoum, Sudan

**Keywords:** left ventricular pseudoaneurysms, percutaneous coronary intervention, computed tomography angiography, cardiac magnetic resonance, case report

## Abstract

Left ventricular pseudoaneurysms (LVPA) are rare complications of myocardial infarction (MI), occurring in less than 1% of cases. Diagnosis is challenging due to nonspecific symptoms, particularly in large aneurysms. Imaging modalities like echocardiography, CTA, and CMR are vital for identifying LVPA, especially in atypical presentations involving massive aneurysms. We report a 75-year-old male with chest pain, diagnosed with inferior wall MI due to left circumflex artery occlusion and treated with PCI. Four weeks later, echocardiography detected an aneurysmal outpouching, confirmed by CTA and CMR, which revealed an unusually large aneurysm with a 30-mm neck, thrombus formation, and extensive myocardial damage. Conservative management was chosen after the patient declined surgery. This case is significant due to the aneurysm’s extraordinary size, emphasizing the role of multimodal imaging, particularly CMR, in diagnosis and management. Further research is needed to refine treatment guidelines for such complex cases.

## Introduction

Pseudoaneurysms (PA) occur when a rupture in the heart wall is not entirely open but is instead enclosed by the surrounding pericardium or scar tissue that forms around the rupture [1]. Left ventricular pseudoaneurysms (LVPA) are a rare condition, affecting about 0.1% of patients following a myocardial infarction, with a mortality rate of 10% [2]. A significant proportion of patients with pseudoaneurysms may not exhibit symptoms [3]. Some patients may have nonspecific symptoms including shortness of breath, chest pain, syncope, and palpitations. These symptoms may overlap with other disorders common in the post-MI timeline, making the diagnosis of a pseudoaneurysm challenging [4].

Echocardiography, computed tomography angiography (CTA), and cardiac magnetic resonance (CMR) are regarded as effective noninvasive imaging modalities for diagnosing pseudoaneurysm [5]. Surgery is suggested for symptomatic patients, giant aneurysms, or the risk of rupture [5]. Conservative treatment is considered for asymptomatic patients or those with aneurysms measuring less than 3 cm of dimension [6].

Herein, we present a rare case of a free wall pseudoaneurysm in a 75-year-old male patient, which developed following a myocardial infarction and subsequent percutaneous coronary intervention (PCI).

## Case presentation

**Figure 1:**
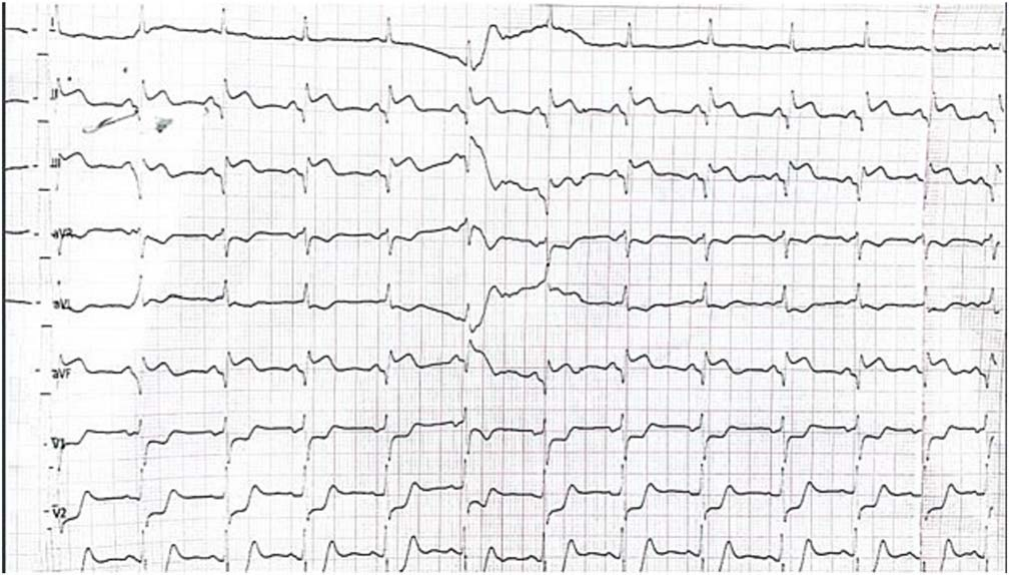
ECG showing ST elevation in inferior limb leads.

A 75-year-old male came late to the hospital after taking PRN antacids for chest pain. Upon arrival, his vital signs were stable, with a heart rate of 78 bpm, blood pressure of 120/70 mmHg, SpO2 of 98% on room air, moreover he has a BMI of 24.57 kg/m^2^. An ECG performed at the clinic indicated an inferior wall myocardial infarction (IWMI) (Fig. 1).

**Figure 2:**
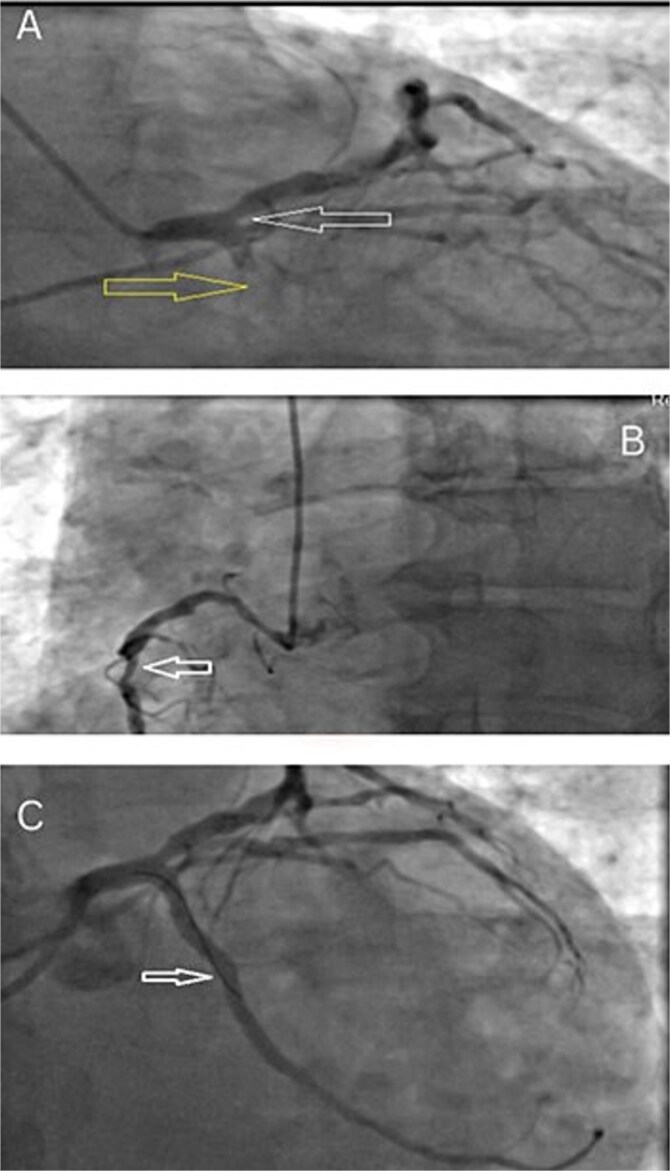
(A) Moderate stenosis is observed in the LAD artery, while complete occlusion of the proximal LCX due to a thrombus. (B) The arrow denotes intermediate disease extending from the proximal to mid segment of the RCA. (C) Post- PCI imaging demonstrates good TIMI flow restoration.

A Transthoracic Echocardiography (TTE) at that time showed a reduced Ejection fraction (EF) of 40% with inferolateral wall hypokinesia. Invasive coronary angiography initially engaged the left coronary system, revealing a complete occlusion of the proximal left circumflex artery (LCX) with distal filling through collateral circulation (Fig. 2A), and Intermediate disease (60–70% stenosis) was resent from the proximal to mid-segment of the right coronary artery (RCA) (Fig. 2B). Meanwhile, percutaneous coronary intervention (PCI) was performed on the LCX using a drug-eluting stent (DES) via right radial access, resulting in TIMI grade 3 flow (Fig. 2C). After the procedure, the radial sheath was removed, and a trans-radial band was applied. The patient was then transferred to the coronary care unit (CCU) for monitoring. The band was deflated after 2–3 h, with the right radial pulse remaining palpable and no complications at the access site. The patient remained stable, reported no discomfort, and was discharged on optimized medical therapy. Four weeks later, at a routine follow-up, the patient remained asymptomatic with good functional capacity (CCS 1 and NYHA 1). Relevant laboratory findings are presented in Tables 1-6.

**Table 1:** Coagulation profile.

Coagulation Profile		
Parameter	Result	Reference Range
PT	12.8	(10–14) sec
INR	1.19	(0.9–1.3) Ratio
APTT	39.4	(26–40) sec

**Table 2:** Renal function test.

Renal Function Test (RFT)		
Parameter	Result	Reference Range
UREA	42	(15–45) mg/dL
Creatinine	1.01	(0.2–1.1) mg/dL
Uric Acid	6.5	(3.4–7.2) mg/dL
BUN	14	(8–22) m

**Table 3:** Lipid profile.

Parameter	Result	Reference Range
Cholesterol	101	(< 200) mg/dL
Triglyceride	187	(< 200) mg/dL
High Density Lipoprotein (HDL)	48	(40–65) mg/dL

**Table 4:** Serum electrolytes (Na, K and Cl).

Parameter	Result	Reference Range
Sodium	135	(136–145) mmol/L
Potassium	4.2	(3.5–5.5) mmol/L
Chloride	104	(98–110) mmol/L

**Table 5:** Cardiac enzyme profile.

Parameter	Result	Reference Range
CPK (Creatine Phosphokinase Test)	49	(0–190) U/L (38–174) U/L
CK-MB	13	(0–25) U/L
LDH	316	(0–480) U/L
AST	40	(0–43) U/L (0–48) U/L

**Table 6:** Complete blood profile.

Parameter	Result	Reference Range
WBC	6.1	(4–10) 10^9/L
RBC	4.81	(3.8–5.5) 10^12/L
GRA	6.8	(1.6–7.6) 10^9/L
LYM	6.8	(1.3–4.5) 10^9/L
Eosinophils	0.1	(0.03–0.64) cells/mcL
MON	1.8	(0.3–1) 10^9/L
Basophils	0.1	(0–0.1) cells/mcL
GR%	43.5	(43.6–75.4) %
LY%	43.9	(16.1–48.5) %
Eosinophils %	0.6	(0.6–7.3) %
MO%	11.6	(4.5–12.1) %
Basophils %	0.4	(0–1.7) %
HGB	13.2	(11.5–16.5) g/dL
HCT	44.4	(35–48.5) %
MCV	92.3	(84–98) fL
MCH	29.6	(27.5–32.4) pg
MCHC	32.1	(31.7–35) g/dL
Rdwc	14.4	(11.1–15) %
Rdws	45.9	(36.2–49.7) fL
PLT	281	(140–450) 10^3/UL
MPV	8.4	(8.3–12.1) fL

**Table 7:** Measurement and calculation of CMR.

Ventricular Measurements & Calculations				
Measurement	Left Ventricle (Measured Value)	Left Ventricle (Standard Range)	Right Ventricle (Measured Value)	Right Ventricle (Standard Range)
Ejection Fraction	35%	56–78%	—%	47–80%
End Diastolic Volume	122 mL	77–195 mL	— ml	58–154 mL
End Systolic Volume	70 mL	19–72 mL	— ml	12–68 mL
Stroke Volume	38 mL	51–133 mL	— ml	35–98 mL
Cardiac Output	3.0 l/min	2.82–8.82 l/min	— l/min	2.82–8.82 l/min
Myocardial Mass	- g	118–238 g	N/A	N/A

**Figure 3:**
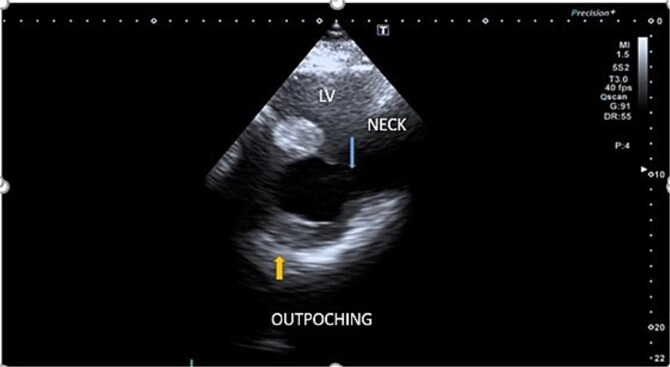
A 2D echocardiogram illustrating an aneurysmal outpouching containing internal echogenic material.

However, a follow-up TTE at 4 weeks revealed moderate left ventricular systolic dysfunction, characterized by hypokinesia of the inferolateral wall and a basal inferior wall aneurysmal outpouching near the anterior mitral leaflet. This outpouching had an approximately 38 mm neck and contained internal echogenic material in apical four chamber view, raising suspicion of a thrombus (Fig. 3). The EF was observed to be 35%–40% (Supplementary Video 1), while the other valves were functioning normally. These findings suggested a possible LVPA; however, due to the large size and wide neck of the outpouching, the echocardiogram was not definitive in confirming the diagnosis.

**Figure 4:**
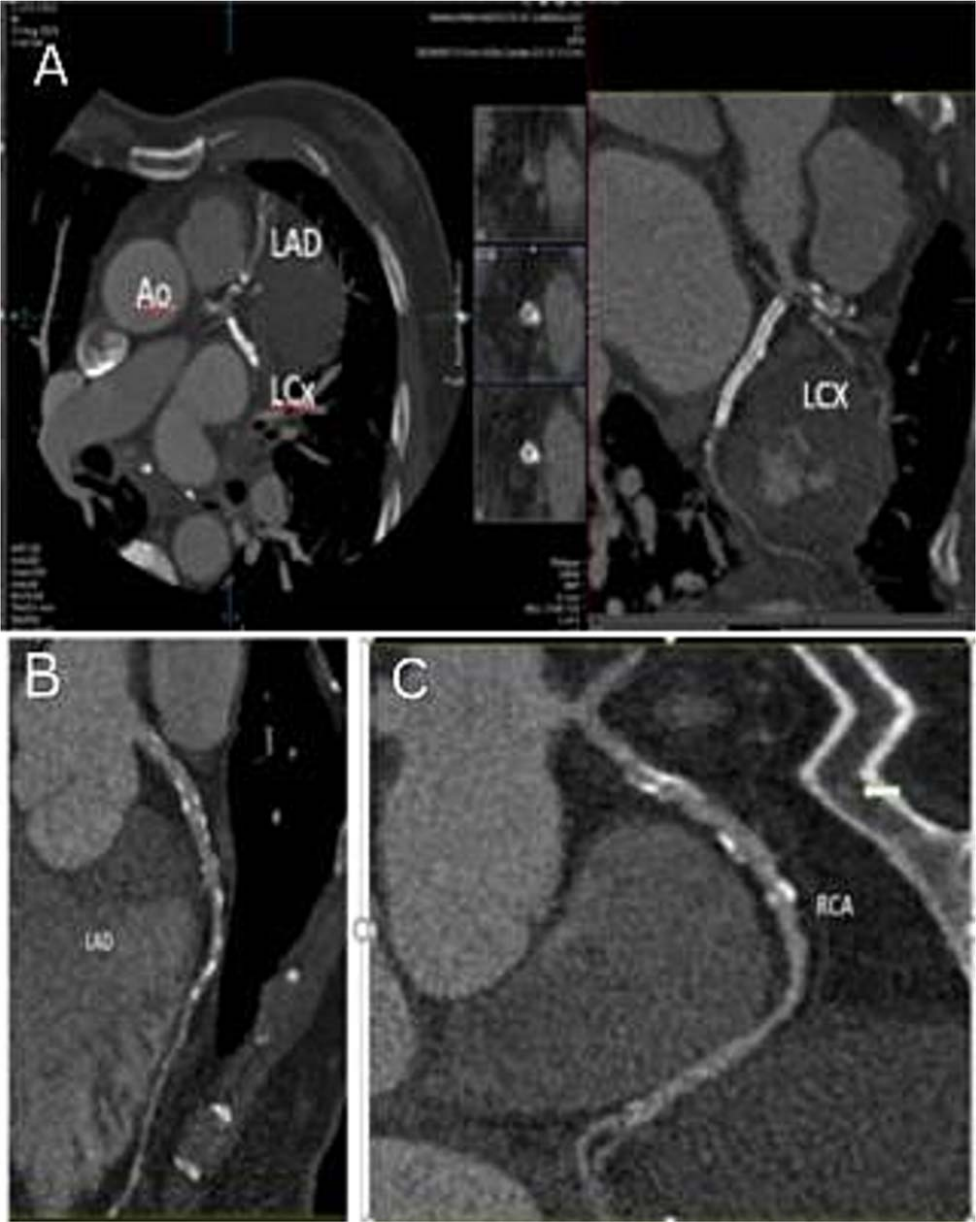
(A) Curved MPR images show a patent stent in the left circumflex artery. (B) the LAD exhibits calcific plaques causing mild stenosis from the proximal to mid-course. (C) the RCA contains calcific plaque resulting in moderate to intermediate stenosis in the mid-course.

**Figure 5:**
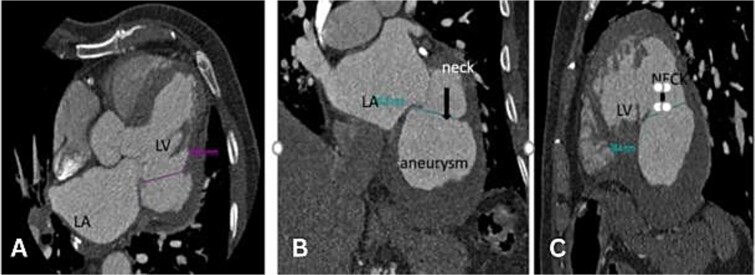
MPR images in axial (A), coronal (B), and sagittal (C) views showing the measurement of the aneurysm neck.

**Figure 6:**
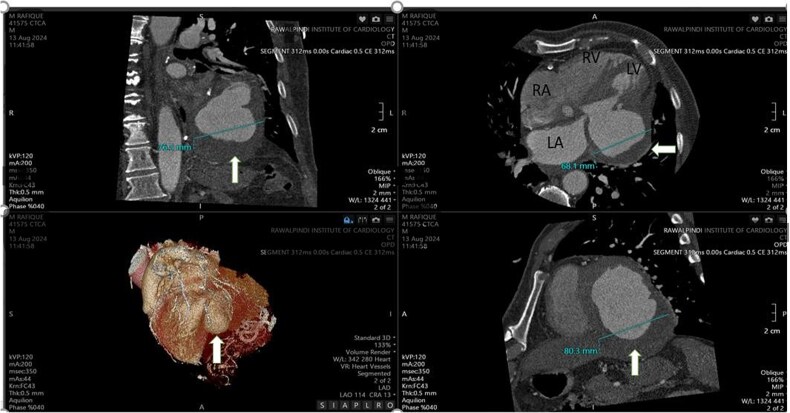
The maximum diameter of the pseudoaneurysm cavity measures 76 mm, 68 mm, and 80 mm across different MPR images shown by white arrow.

A CT angiogram confirmed the patency of the previously placed stent (Fig. 4A). The craniocaudal axial CTA revealed a large contrast-filled outpouching in the basal inferior and inferolateral walls, with an irregular thrombus lining inside (30 to 45 HU) (Supplementary Video 2). Calcified plaque was observed in the LAD and RCA, causing mild stenosis and intermediate stenosis, respectively (Figs 4B and5C). Multiplanar Reconstruction (MPR) images (Fig. 5A-C) measured the aneurysm’s neck at 30 mm, 38 mm, and 36 mm, respectively. The maximum diameter of the aneurysm cavity was also measured in various MPR images, with dimensions of 80 mm, 68 mm, and 76 mm (Fig. 6).

**Figure 7:**
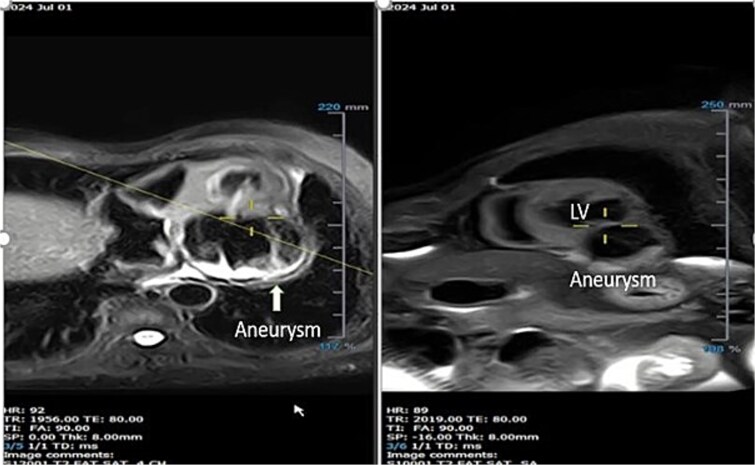
T2-weighted imaging stills focusing on the LV aneurysm in long-axis (a) and short-axis (b) views, showing diffuse hyperintense signals throughout the aneurysmal wall.

**Figure 8:**
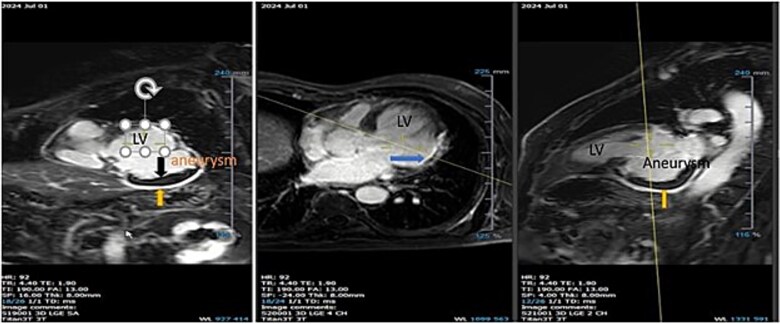
Late gadolinium enhancement images—Short-axis view (SAX) (a), 4-chamber long-axis view (4CH LAX) (b), and 2-chamber long-axis view (2CH LAX) (c)—Demonstrate homogeneous hyperenhancement of the entire outer layer of the aneurysm suggestive of inflammation.

T2-weighted images focusing on the aneurysm in long-axis (Fig. 7A) and short-axis (Fig. 7B) views showed diffuse hyperintense signals throughout the aneurysmal wall, indicative of active edema. First-pass perfusion images demonstrated normal myocardial perfusion from the apex to the base, with hypoperfused (dark) areas along the entire lining of the aneurysm (Supplementary Video 4). LGE images, including short-axis (Fig. 8A), 4-chamber long-axis (Fig. 8B), and 2-chamber long-axis (Fig. 8C) views, showed homogeneous hyperenhancement of the aneurysm’s outer layer (indicated by the yellow arrow), suggesting an inflamed pericardium (Supplementary Video 5). The remaining myocardial segments exhibited normal thickness and function. The pattern of LGE made it possible to clearly characterize the tissue layers of the aneurysmal outpouching as pericardium and thrombus. The pericardial enhancement was smooth, homogeneous, and shiny, while the myocardial LGE had a slightly nonhomogeneous, granular appearance, even if it was transmural.

With these findings, a definitive diagnosis of LVPA was made. The patient was offered surgical intervention and guideline-directed medical management, including anticoagulation with lifelong Rivaroxaban and Clopidogrel. However, after a thorough discussion of the risks and benefits, the patient declined surgical repair of the pseudoaneurysm and opted for close clinical follow-up with plans for surveillance by the cardiothoracic surgery department.

## Discussion

This case highlights a rare but significant complication following myocardial infarction and PCI. Although PA of the left ventricle is uncommon, with an incidence of less than 1% post-MI and even lower in patients undergoing PCI, their diagnosis remains challenging due to frequently asymptomatic presentations [7]. In our case, the rupture involved the inferior wall, deviating from the more common inferolateral and posterior rupture sites.

A multimodal imaging approach was essential for accurate diagnosis and characterization. Initial TTE identified the pseudoaneurysm, aligning with its role as a first-line imaging modality for post-MI complications [8]. However, TTE alone has limitations due to poor acoustic windows, making it difficult to assess aneurysm extent and myocardial integrity [9]. CTA provided further anatomical clarity and confirmed stent patency, supporting its complementary role in pseudoaneurysm assessment. CMR, with its superior spatial resolution and tissue characterization, was crucial in differentiating the pseudoaneurysm from a true aneurysm. CMR revealed extensive thrombus formation and a full-thickness myocardial rupture, reinforcing its value in evaluating complex cardiac anomalies.

LVPA typically results from a contained myocardial rupture and can develop due to various factors, including delayed reperfusion, infarct expansion, mechanical injury, or pre-existing myocardial damage [10]. Our patient’s delayed presentation to the hospital likely contributed to infarct expansion, predisposing him to pseudoaneurysm formation. Additionally, the possibility of a prior silent MI causing myocardial weakening cannot be ruled out.

Management strategies for LVPA depend on aneurysm size, symptoms, and rupture risk. Literature suggests that asymptomatic pseudoaneurysms smaller than 3 cm may be managed conservatively with close monitoring [11]. However, surgical intervention is often recommended regardless of size due to the potential risk of rupture and sudden death [12]. Despite the surgical recommendation, our patient opted for conservative management after an informed discussion of the risks and benefits. This case underscores the importance of individualized management decisions and highlights the role of advanced imaging in optimizing patient outcomes.

## Conclusion

This case underscores the critical role of a multimodal imaging approach in the diagnosis and management of pseudoaneurysms. The integration of echocardiography, CTA, and CMR provided a comprehensive assessment, enabling precise diagnosis and guiding appropriate intervention. Given the rarity and potential severity of pseudoaneurysms, ongoing vigilance with structured follow-up is essential to ensure timely detection of complications and optimize patient outcomes.

Future research should focus on refining imaging protocols to enhance early detection and risk stratification. Additionally, studies exploring standardized follow-up strategies, long-term outcomes of different treatment approaches, and the role of emerging imaging techniques could further improve clinical decision-making. Comparative analyses of surgical versus conservative management, including patient selection criteria and long-term prognosis, are needed to establish clearer guidelines. Investigating novel biomarkers and computational modeling may also aid in identifying high-risk patients, ultimately advancing personalized treatment strategies for this complex condition.

## Supplementary Material

Supplementary_Video_1_omaf088

Supplementary_Video_2_omaf088

Supplementary_Video_3_omaf088

Supplementary_Video_4_omaf088

Supplementary_Video_5_omaf088

## References

[1] Frances C, Romero A, Grady D. Left ventricular pseudoaneurysm. J Am Coll Cardiol 1998;32:557–61. 10.1016/S0735-1097(98)00290-3 9741493

[2] Faiza Z, Lee LS. Left Ventricular False Aneurysm Zainab Faiza. [Updated 2022 Sep 19. In: StatPearls [Internet]. StatPearls: Treasure Island (FL). 31855363

[3] Yeo TC, Malouf JF, Oh JK. et al. Clinical profile and outcome in 52 patients with cardiac Pseudoaneurysm. Ann Intern Med 1998;128:299–305. 10.7326/0003-4819-128-4-199802150-00010 9471934

[4] Gupta S, Ankush A, Gandhi P. et al. Cardiac MRI in the diagnosis and management of left ventricular pseudoaneurysms with previous myocardial infarction: a report of two cases. Radiol Case Rep 2024;19:4242–7. 10.1016/j.radcr.2024.07.007 39135675 PMC11318547

[5] Prêtre R, Linka A, Jenni R. et al. Surgical treatment of acquired left ventricular pseudoaneurysms. Ann Thorac Surg 2000;70:553–7. 10.1016/S0003-4975(00)01412-0 10969679

[6] Mujanovic E, Bergsland J, Avdic S. et al. Surgical treatment of left ventricular Pseudoaneurysm. Med Arch 2014;68:215–7. 10.5455/medarh.2014.68.215-217 25568538 PMC4240329

[7] Hobbs RD, Assi A, Bolling SF. et al. Long-term survival and echocardiographic findings after surgical ventricular restoration. Ann Thorac Surg 2019;107:1754–60. 10.1016/j.athoracsur.2018.11.054 30586580

[8] Khattar RS, Senior R. Echocardiography. In: Chronic Coronary Artery Disease. Elsevier; 2018. p. 128–46. 10.1016/B978-0-323-42880-4.00011-X.

[9] Kaur N, Panda P, Choudhary AK. et al. Left ventricular pseudoaneurysm: imaging. BMJ Case Rep 2021;14:e243913. 10.1136/bcr-2021-243913 PMC8220522 34158338

[10] Lorusso R, Cubeddu RJ, Matteucci M. et al. Ventricular Pseudoaneurysm and Free Wall rupture after acute myocardial infarction. J Am Coll Cardiol 2024;83:1902–16. 10.1016/j.jacc.2023.10.054 38719370

[11] Rivera PA, Dattilo JB. Pseudoaneurysm. 2024 Feb 17. In: StatPearls [Internet]. Treasure Island (FL): StatPearls Publishing; 2025 Jan–. PMID: 31194401. 31194401

[12] Zhong Z, Song W, Zheng S. et al. Surgical and conservative treatment of post-infarction left ventricular Pseudoaneurysm. Front Cardiovasc Med 2022;9:9. 10.3389/fcvm.2022.801511 PMC8829002 35155628

